# Supplementary material for: Conditional chemoconnectomics (cCCTomics) as a strategy for efficient and conditional targeting of chemical transmission
Source: eLife. 2024 Apr 30;12:RP91927. doi: 10.7554/eLife.91927 (PMC11060718; doi:10.7554/eLife.91927)
Supplement: Figure 3—source data 3. [file elife-91927-fig3-data3.zip › Figure 3 source data3 part 2.zip!/Figure 3-Source data 3-part2/1053f2-1-CRISPResso_Report_y3hlioj1.zip!/CRISPResso_on_1053f2_2/1a.Read_barplot.pdf]

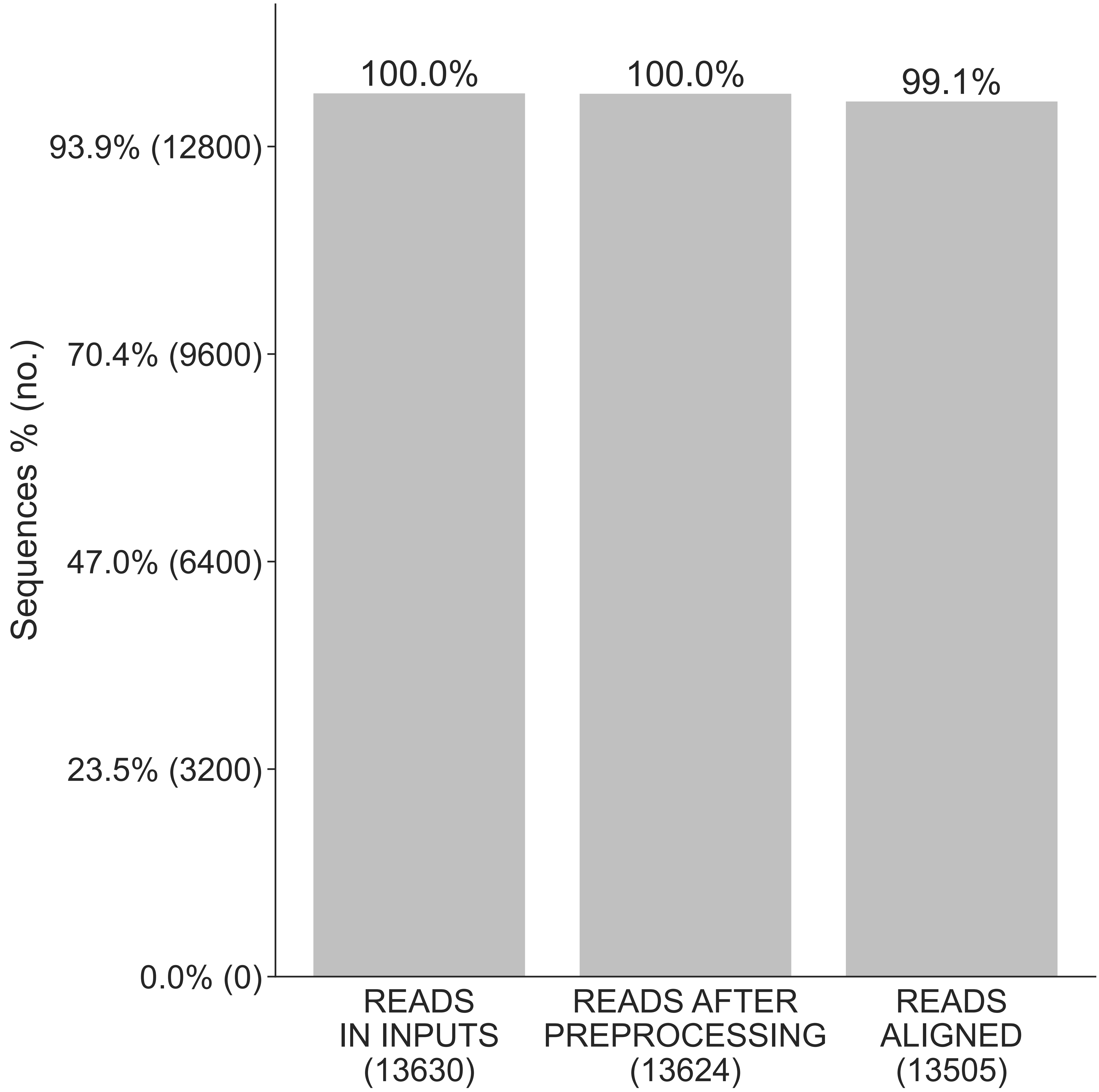

Sequences % (no.)
93.9% (12800)
70.4% (9600)
47.0% (6400)
23.5% (3200)
0.0% (0)
100.0%
100.0%
99.1%
READS IN INPUTS (13630)
READS AFTER PREPROCESSING (13624)
READS ALIGNED (13505)
